# Supplementary material for: Transmission of tuberculosis between foreign-born and Finnish-born populations in Finland, 2014–2017
Source: PLoS One. 2021 Apr 23;16(4):e0250674. doi: 10.1371/journal.pone.0250674 (PMC8064540; doi:10.1371/journal.pone.0250674)
Supplement: S1 Fig — The tree was generated with Bionumerics 6.6 using the UPGMA (unweighted pair group method with arithmetic mean) method on the categorical values of the similarity matrix of the MIRU-VNTR results. (PDF) [file pone.0250674.s001.pdf]

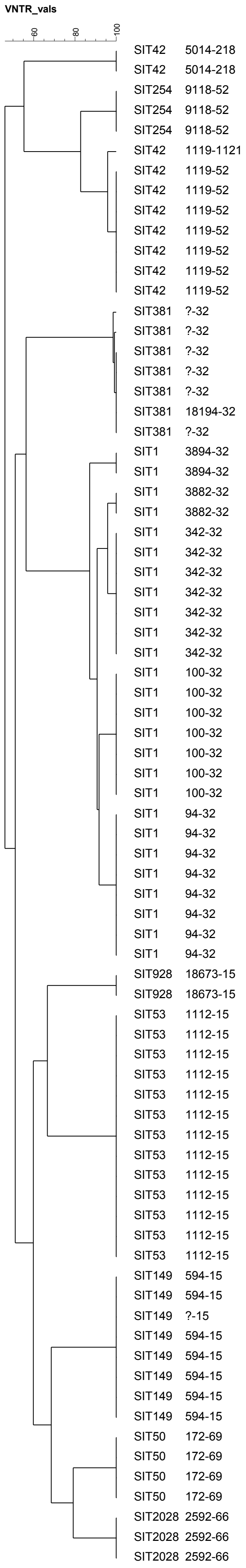

S1 Fig. Phylogenetic tree of the mixed clusters. The tree was generated with Bionumerics 6.6 using the UPGMA (unweighted pair group method with arithmetic mean) method on the categorical values of the similarity matrix of the MIRU-VNTR results.
