# Supplementary material for: Transmission of tuberculosis between foreign-born and Finnish-born populations in Finland, 2014–2017
Source: PLoS One. 2021 Apr 23;16(4):e0250674. doi: 10.1371/journal.pone.0250674 (PMC8064540; doi:10.1371/journal.pone.0250674)
Supplement: Attachment — Submitted filename: Response to Reviewers.pdf [file pone.0250674.s002.pdf]

Dear Editor,

Thank you for the positive response on our manuscript entitled " Transmission of tuberculosis between foreign-born and Finnish-born populations in Finland, 2014-2017" (Manuscript ID PONE-D-20-38987). We have to the best of our ability approached and answered the reviewer's and editor's comments and questions. We hope that our manuscript is now suitable for publication in your journal. In the revised manuscript, Figures and Tables, we have indicated changes in the text with "Track changes".

Please find our answers and input below.

Reviewer #1:

The manuscript entitled "Transmission of tuberculosis between foreign-born and Finnish-born populations in Finland, 2014-2017" describes the epidemiology of tuberculosis and evaluates the disease transmission between foreign-born and Finnish-born populations using MIRU-VNTR. While the study attempts to understand the major concerns on TB transmission in Finland, it cannot be accepted in its current form and requires following revisions.

Minor comments:

1. There are inconsistencies in writing throughout the manuscript. Mycobacterium tuberculosis is written as M. tuberculosis, MTB and Mycobacterium tuberculosis. MTB abbreviation has not been described anywhere

   Answer: Thank you for your observation. The inconsistencies are now revised and the writing has been made consistent throughout the manuscript. MTB abbreviation has been changed to M. tuberculosis. All the changes are shown in tracked changes in the manuscript.

2. MIRU-VNTR was first used in abstract (line 25). However, the abbreviation was first described in line number 75 under methods

   Answer: Thank you for your observation. In abstract, lines 27-28, MIRU-VNTR has been changes to Mycobacterial Interspersed Repetitive Unit Variable Number Tandem Repeat.

   Also a change has been made to the methods section, lines 78-80: Culture-positive M. tuberculosis isolates are routinely sent to the THL Mycobacterial Reference Laboratory for drug susceptibility testing and genotyping by spoligotyping and Mycobacterial Interspersed Repetitive Unit Variable Number Tandem Repeat (MIRU-VNTR) (24 loci) according to standard protocols [14, 15].

3. In line 85 “The genotypes of the mixed clusters were 85 compared with a local genotyping database”- Please provide the link or reference to the database

   Answer:Thank for the comment. Unfortunately there is no link or reference to the local genotyping database. The data can be accessed by applying for a data permit through the Health and Social Data Permit Authority (Findata) (https://www.findata.fi/en/).

4. A figure describing close genetic relatedness between epidemiological risk factors (immigration status, age, history of TB) and MIRU-VNTR profiles will help in data interpretation

   Answer: We agree with the reviewer that this kind of figure might help with the data interpretation in some circumstances. However, presenting the odds ratios for different epidemiologic risk factors graphically is not feasible because of small number (n=76) of isolates in mixed clusters in our study and would not enable drawing conclusions from the data. Whole genome sequencing (WGS) with thorough data analysis and contact tracing would be more feasible method to get detailed information about the genetic relatedness.

5. Please describe line number 72- “additional information on the contact tracing (free text)”

   Answer: Thank you for the clarifying question. By free text we mean a free text column where phycisians are able to add additional information regarding the contact tracing.

   Sentence is revised in the methods section, line 75: free text <u>column on the notification form</u>

6. Under methods section, please include information on the kind of culture technique used (liquid or solid?). Considering the possibility that an individual may be infected with multiple strains, it is important to mention if the samples for DNA extraction were taken from a single or multiple colony

   Answer: Thank you for a valid point. We have included information regarding culture techniques used.

   Sentence added in the methods section, lines 81-83: DNA was extracted from bacterial colonies grown on solid medium using the CTAB (cetyl trimethylammonium bromide) method [16, 17].

7. Please include the DNA extraction method (kit and reagent information or a reference to the previously described method)

Answer: We have now included the DNA extraction method used.

The first paragraph of the laboratory methods and identification of clusters revised, lines 79-86: Culture-positive M. tuberculosis isolates are routinely sent to the THL Mycobacterial Reference Laboratory for drug susceptibility testing and genotyping by spoligotyping and Mycobacterial Interspersed Repetitive Unit Variable Number Tandem Repeat (MIRU-VNTR) (24 loci) according to standard protocols [14, 15]. DNA was extracted from bacterial colonies grown on solid medium using the CTAB (cetyl trimethylammonium bromide) method [16, 17]. Spoligotyping was performed using spoligotyping membranes obtained from Ocinum Biosolutions (Hyderabad, P.A., India) or produced in-house as previously described by Kamerbeek et al. [15]. The MIRU-VNTR analysis was performed at GenoScreen (Lille, France) using the MIRU-VNTR Kits of the same company.

Also, two references were added and the reference list revised accordingly:

16. van Soolingen D, Hermans PW, de Haas PE, Soll DR, van Embden JD. Occurrence and stability of insertion sequences in Mycobacterium tuberculosis complex strains: evaluation of an insertion sequence-dependent DNA polymorphism as a tool in the epidemiology of tuberculosis. J Clin Microbiol. 1991;29(11):2578-2586. doi:10.1128/JCM.29.11.2578-2586.1991

17. van Beek J, Haanperä M, Smit PW, Mentula S, Soini H. Evaluation of whole genome sequencing and software tools for drug susceptibility testing of Mycobacterium tuberculosis. Clin Microbiol Infect. 2019;25(1):82-86. doi:10.1016/j.cmi.2018.03.041

8. Please provide information on which method was used for MIRU-VNTR typing (15 or 24-locus). Include MIRU-VNTR typing spoligotyping kit information

   Answer: Thank you for the clarifying point. We have used 24 loci MIRU-VNTR and also kit information added. See added paragraph above.

9. It is not cleared how cluster cutoff was decided in MIRU-VNTR. For example, do you call the . isolates as clusters if 24 loci matched? How do you interpret the samples with mixed MIRU-VNTR patterns?

   Answer: Thank you for this question. In our study clusters were defined based on both MIRU-VNTR and spoligotyping. Both (MIRU-VNTR and spoligotyping) needed to match so that a cluster was defined. As mentioned in the manuscript on the "Laboratory methods and identification of clusters" section: "When two or more M. tuberculosis isolates matched by

genotyping methods (i.e., identical spoligotype and MIRU-VNTR patterns), they were considered a genotype cluster." That said, if only MIRU-VNTR was the same with two (or more) isolates, it was not considered as a cluster.

10. A phylogenetic tree could be constructed based on MIRU-VNTR typing data for better visualization of relatedness among the samples.

    Answer: A phylogenetic tree has been added as a supplementary file due to size of the tree.

    Also, a sentence added in the methods section, lines 92-94: Clustering of isolates with incomplete MIRU-VNTR profile due to missing/undetectable, double, or non-numerical (e.g. 3s) results was performed with help of UPGMA (unweighted pair group method with arithmetic mean) tree generated in the Bionumerics software.

    Sentece added in the results section, lines 148-149: A phylogenetic tree was constructed based on MIRU-VNTR typing data to show the relatedness among mixed clusters (S1 Fig).

When submitting your revision, we need you to address these additional requirements.

1. Please ensure that your manuscript meets PLOS ONE's style requirements, including those for file naming. The PLOS ONE style templates can be found at https://journals.plos.org/plosone/s/file?id=wjVg/PLOSOne_formatting_sample_main_body.pdf and https://journals.plos.org/plosone/s/file?id=ba62/PLOSOne_formatting_sample_title_authors_affiliations.pdf

Answer: We have ensured that our manuscript meets PLOS ONE's style requirements.

2.We note that you have indicated that data from this study are available upon request. PLOS only allows data to be available upon request if there are legal or ethical restrictions on sharing data publicly. For information on unacceptable data access restrictions, please see http://journals.plos.org/plosone/s/data-availability#loc-unacceptable-data-access-restrictions.

In your revised cover letter, please address the following prompts:

a) If there are ethical or legal restrictions on sharing a de-identified data set, please explain them in detail (e.g., data contain potentially identifying or sensitive patient information) and who has imposed them (e.g., an ethics committee). Please also provide contact information for a data access committee, ethics committee, or other institutional body to which data requests may be sent.

Answer: We wish we could be providing the data set, but our data includes sensitive patient information. However, the Finnish Communicable Diseases Act 1227/2016, 42 § states the following: Release of information for research The Finnish Institute for Health and Welfare

may, notwithstanding confidentiality provisions, decide to release personal data from registers referred to in this Act that are maintained by the institute, if the release is made for the purpose of scientific research on health care activities, prevention or treatment of diseases, or research related to these, and if the release meets the requirements laid down in section 28 of the Act on the Openness of Government Activities (621/1999).
That said, the data permit can be applied for scientific research through the Health and Social Data Permit Authority (Findata) (https://www.findata.fi/en/)

b) If there are no restrictions, please upload the minimal anonymized data set necessary to replicate your study findings as either Supporting Information files or to a stable, public repository and provide us with the relevant URLs, DOIs, or accession numbers. Please see http://www.bmj.com/content/340/bmj.c181.long for guidelines on how to de-identify and prepare clinical data for publication. For a list of acceptable repositories, please see http://journals.plos.org/plosone/s/data-availability#loc-recommended-repositories.

We will update your Data Availability statement on your behalf to reflect the information you provide.

3. Your ethics statement should only appear in the Methods section of your manuscript. If your ethics statement is written in any section besides the Methods, please move it to the Methods section and delete it from any other section. Please ensure that your ethics statement is included in your manuscript, as the ethics statement entered into the online submission form will not be published alongside your manuscript.

Answer: Ethical statement was moved into the Methods section

4.We noticed you have some minor occurrence of overlapping text with the following previous publications, which needs to be addressed:
- https://www.cambridge.org/core/journals/epidemiology-and-infection/article/tuberculosis-in-immigrants-in-finland-19952013/7ED2C7F8A951802B8722AB06D552285B
- https://bmcpublichealth.biomedcentral.com/articles/10.1186/s12889-018-5501-y
In your revision ensure you cite all your sources (including your own works), and quote or rephrase any duplicated text outside the methods section. Further consideration is dependent on these concerns being addressed.

Answer: Thank you for checking out the overlapping text with previous studies. These two studies mentioned here are our own articles and the methods are same or similar. Other parts have been cited accordingly.
